# LDH@Boronate Polymer Core–Shell Nanoparticles: Nanostructure Design for Synergistically Enhancing the Flame Retardancy of Epoxy Resin

**DOI:** 10.3390/polym15092198

**Published:** 2023-05-05

**Authors:** Cheng Chi, Siyuan He, Chaohua Peng, Birong Zeng, Long Xia, Zhongxi Miao, Hui Xu, Shuchuan Wang, Guorong Chen, Lizong Dai

**Affiliations:** 1 Fujian Provincial Key Laboratory of Fire Retardant Materials, College of Materials, Xiamen University, Xiamen 361005, China; iserlohner@foxmail.com (C.C.); 20720201150052@stu.xmu.edu.cn (S.H.); 20720200155774@stu.xmu.edu.cn (C.P.); 20720190153839@stu.xmu.edu.cn (L.X.); 20720201150122@stu.xmu.edu.cn (Z.M.); 20720201150095@stu.xmu.edu.cn (H.X.); grchen@xmu.edu.cn (G.C.); 2 Xiamen Key Laboratory of Fire Retardant Materials, College of Materials, Xiamen University, Xiamen 361005, China; 3 T&H Novel Materials Co., Ltd., Quanzhou 362000, China; wangsc@thnm.com

**Keywords:** layered double hydroxides, flame retardancy, core–shell structure, epoxy resin, boronate polymer, nanocomposites

## Abstract

As a promising nanofiller, layered double hydroxides (LDHs) can advance the fire safety of epoxy resin (EP), but so far, due to the problems of dispersion and low efficiency, it has still been a challenge to incorporate the flame retardancy and mechanical properties of EP nanocomposites effectively under the circumstance of a low additive amount. In this work, we take LDHs as the template, via the adsorption of a catechol group and the condensation polymerization between catechol groups and phenylboric acid groups, to prepare a core–shell structured nanoparticle LDH@BP, which contains rich flame-retardant elements. EP/LDH@BP nanocomposites were prepared by introducing LDH@BP into EP. The experimental results indicate that, compared with the original LDH, LDH@BP disperses uniformly in the EP matrix, and the flame retardancy and mechanical properties of EP/LDH@BP are significantly improved. At a relatively low content (5 wt%), EP/LDH@BP reached the rating of V-0 in the UL-94 test, LOI was increased to 29.1%, and peak heat release rate (PHRR) was reduced by 35.9% in cone calorimeter tests, which effectively inhibited the release of heat and toxic smoke during the combustion process of EP. Simultaneously, the mechanical properties of EP/LDH@BP have been improved satisfactorily. The above results derive from the reasonable architectural design of organic–inorganic nano-hybrid flame retardants and provide a novel method for the construction of efficient and balanced EP nanocomposite system with LDHs.

## 1. Introduction

With the continuous development and evolution of polymer materials, epoxy resin (EP) is extensively applied in many vital fields because of its excellent mechanical properties, chemical resistance and dimensional stability [1,2,3]. Meanwhile, the flammable character of EP also brings some hidden dangers, which pose a significant threat to the safety of people’s life and property and greatly limit EP’s application [4,5,6]. Therefore, it is of great urgency to design novel EP composites with excellent fire resistance.

Currently, nanoparticle flame retardants directly incorporated into the EP matrix show great potential, possessing a convenience of use and wide design space [7]. Typically, nanoparticle flame retardants are based on inorganic nanomaterials (e.g., graphene and its derivatives, hexagonal boron nitride, metal organic frameworks, polyhedral oligomeric silsesquioxane and layered double hydroxides, etc.) [8,9,10,11,12], and they have significant advantages in reducing heat release and smoke production during the combustion process of EP. However, untreated inorganic flame retardants usually show limited flame retardancy efficiency, which makes it difficult for a low amount to enable EP nanocomposites to achieve the rating of the UL-94 vertical burning tests. Meanwhile, a high loading of nanofillers tends to adversely affect the mechanical properties and dispersion of nanocomposites [13,14]. In previous studies, organic–inorganic nano-hybrid flame retardants represented a corresponding strategy to modify inorganic nanoparticles with organic molecules and integrate flame-retardant elements such as P, N, and B into the system, which can endow nanoparticles with excellent flame-retardant properties and improve their interface interaction with the EP matrix [15,16]. Apparently, rational selection of organic components and inorganic nanoparticles combined with appropriate system design is the necessary condition for the construction of efficient organic–inorganic nano-hybrid flame retardants.

Layered double hydroxides (LDHs) are a class of two-dimensional nanomaterials with a layered structure, which are composed of positively charged metal laminates, interlayer anions, and solvated molecules [17]. In recent years, LDHs have been widely used in catalysts, adsorbents, membranes, supercapacitors, flame retardants, and other fields [18,19,20,21,22]. As excellent nanofillers, LDHs are able to undergo endothermic reaction and form bimetallic oxide as a physical barrier layer to block external heat at high temperature. At the same time, they can release interlayer water molecules and carbon dioxide to dilute combustible gas, which effectively inhibits the thermal degradation of polymer in the combustion process [23,24]. Therefore, LDHs are regarded as a kind of promising environmentally friendly flame retardant. For the EP nanocomposites, the dispersion state of nanofillers is very important to the comprehensive properties of materials. However, due to the hydrophilicity of LDHs, the strong van der Waals force among layers, and the poor compatibility with polymer, LDHs are prone to agglomeration in the matrix, which harmfully influences the flame retardancy and mechanical properties of EP nanocomposites [25,26]. To solve the above problems, scholars usually use some functional anions to enlarge the basal spacing between the laminates, to improve the stacking phenomenon of LDHs [27,28,29]. Alternatively, they modify the surface of LDHs by the method of chemical grafting or electrostatic adsorption, to enhance the compatibility and dispersion of LDHs in the EP matrix [30,31,32]. However, on the whole, for LDHs, due to the characteristics of an uncontrollable agglomeration tendency, unsatisfactory flame retardancy, poor compatibility with the matrix, and the difficulty of participating in the curing crosslinking procedure of EP, there exist some dilemmas that need further exploration by researchers. Hence, it is still a challenge to properly design a system of organic–inorganic nano-hybrid flame retardants and subtly modify LDHs with appropriate organic molecules, to efficiently improve their interface interaction in the EP matrix, and advance the comprehensive performance of EP nanocomposites at the same time.

At present, there is no report on coating LDHs with polymer shells as flame retardants by the facile condensation method. Based on the above discussion, we developed a novel strategy to prepare an organic–inorganic nano-hybrid flame retardant by coating LDHs with boronate polymer (BP), which provides a convenient idea for regulating the interface interaction among nanoparticles and the EP matrix and endowing the nanoparticles with the expected function. In brief, we prepared MgAl-LDH by the hydrothermal method and used them as the core for flame-retardant nanoparticles. Based on the excellent flame-retardant efficiency of the phosphonitrile structure, 9, 10-Dihydro-9-oxa-10-phosphaphenanthrene-10-oxide (DOPO), and their derivatives, we introduced them into the system. The multi-armed catechol monomers and two-armed phenylboric acid monomers containing rich flame-retardant elements were prepared by the Schiff base reaction and Kabachnik–Fields reaction. Then, through the adsorption of catechol groups on the surface of LDHs and the condensation polymerization between catechol and phenylboric acid, we obtained LDHs coated with boronate polymer (denoted as LDH@BP). Finally, we applied LDH@BP to the preparation of EP nanocomposites, evaluated the effects of LDH@BP on the flame retardancy, mechanical properties and interfacial interactions of EP nanocomposites, and investigated its functional mechanism from the perspective of the condensed phase and gas phase.

## 2. Experimental Section

### 2.1. Materials

The related chemical materials and solvents in this work were obtained for direct use and without further purification, and the details covered in this section can be found in the supporting information.

### 2.2. Monomer Synthesis

**Scheme 1:**
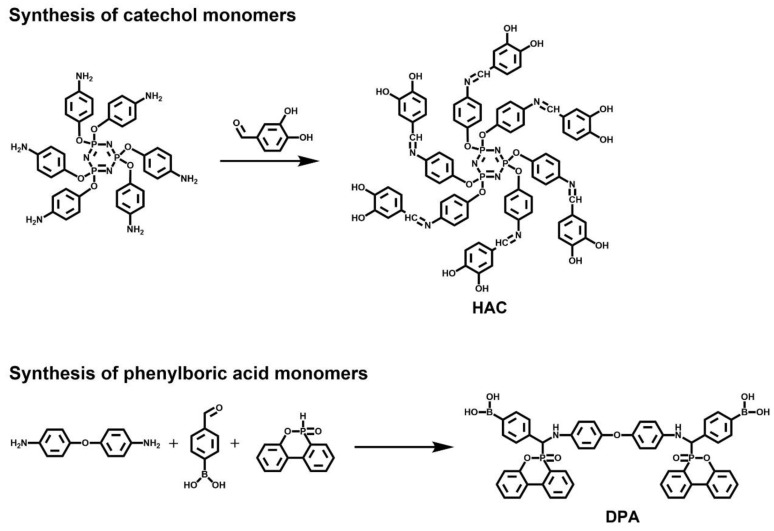
The synthetic route of HAC and DPA.

Based on the Schiff base reaction and Kabachnik–Fields reaction, we synthesized multi-armed catechol monomers and two-armed phenylboric acid monomers, respectively. The main synthesis process and the chemical structures are shown in Scheme 1, and the detailed synthesis procedures can be found in the supporting information.

### 2.3. Preparation of MgAl-LDH

MgAl-LDH nanosheets were prepared by the hydrothermal method [33]. Typically, 3 mmol Mg(NO_3_)_2_·6H_2_O and 1 mmol Al(NO_3_)_3_·9H_2_O were completely dissolved in 10 mL deionized water, then rapidly added to 40 mL NaOH solution (0.15 M) and stirred at room temperature for 10 min. LDH slurry was obtained by centrifugation and washed with deionized water. Then, the slurry was dispersed in deionized water and transferred to a Teflon stainless steel autoclave for hydrothermal reaction at 10 °C for 12 h. After the hydrothermal reaction, MgAl-LDH nanosheets were obtained by centrifugation and freeze-drying.

### 2.4. Preparation of LDH@BP Nanoparticles

A quantity of 100 mg MgAl-LDH nanosheets were dispersed in 50 mL anhydrous methanol under the condition of sonication. Then, 10 mL of absolute methanol solution containing HAC (10 mg/mL) was added to it. After the 60 min sonication treatment, 18 mL absolute methanol solution containing DPA (10 mg/mL) was slowly added to the system under stirring conditions and reacted for 12 h. The product was obtained by centrifugation, washed with absolute methanol, and dried in a vacuum.

### 2.5. Preparation of EP/LDH@BP Nanocomposites

The EP/LDH@BP nanocomposites were prepared by the thermal curing of DGEBA with the curing agent DDM and LDH@BP nanoparticles. For instance, the sample EP/LDH@BP5 was prepared by the following steps: 2.5 g LDH@BP was completely dispersed in 20 mL dichloromethane under the condition of sonication and mixed with 38 g DGEBA. Subsequently, under stirring conditions, dichloromethane was totally removed by vacuum at 80 °C, and 9.5 g DDM curing agent was added to the mixture. Finally, EP/LDH@BP nanocomposites were treated with a three-step curing procedure: 120 °C/4 h, 140 °C/2 h, and 180 °C/2 h. As the control group, EP/LDH/BP was prepared by adding 5 wt% LDH/BP (in the form of physical blending; the mass ratio of LDH:BP was 26.3:73.7). Similarly, other control groups were prepared through the same process (Table S1).

### 2.6. Characterization

Scanning electron microscopy (SEM, SU-70, Japan Hitachi Hi-tech Nagase Office, Japan) was used to obverse the morphologies of LDH, LDH@BP, the fracture surfaces of EP nanocomposites, and the char residues of EP nanocomposites. Transmission electron microscopy (TEM, JEM-2100, JEOL, Japan) was used to investigate the microstructure of LDH, LDH@BP. The high-angle annular dark-field (HAADF) and the elemental energy-dispersive X-ray spectroscopy (EDX) mapping of LDH@BP were investigated with an FEI Talos F200 microscope. Fourier transform infrared (FTIR) spectra were obtained on a Nicolet Avatar 360 spectrophotometer (Thermo Fisher, Waltham, MA, US), in the range of 500–4000 cm^−1^. X-ray photoelectron spectroscopy (XPS) was performed by using a K-Alpha equipped with an Al Kα radiation source (Thermo Fisher, US), and the data were analyzed by Avantage. X-ray diffraction (XRD) patterns were obtained with a Bruker-axs D8-A25 (Bruker, Zurich, Switzerland) with Cu Kα radiation (λ = 1.54178 Å), in the 2θ range of 5–90°. A HORIBA Xplora Raman spectrometer (Horiba, Kyoto, Japan) was used to acquire the Raman spectra of char residues of EP and other EP nanocomposites. The nuclear magnetic resonance (^1^H and ^31^P, NMR) spectra of HNCP, HACP, HAC and DPA were recorded with a Bruker Advanced II AV500 MHz NMR spectrometer (Bruker, Switzerland), with solvent DMSO-d6. 

The flame retardancy of EP nanocomposites was examined with the UL-94 vertical burning tests (ASTM D 3801 standard), limiting oxygen index (GB/T2406-93 standard, with JF-3 instrument), and cone calorimeter test (ISO 5660 criterion, at 35 kW m^−2^ heat flux). TGA-FTIR results were obtained with the PE STA 6000-Frontier instrument (PE, US) under a nitrogen atmosphere, at the heating rate of 10 °C/min from 40 °C to 850 °C. The thermal stability of samples was investigated with a Netzsch STA 409EP thermogravimetric analyzer (Netzsch, Germany) under a nitrogen atmosphere, at the heating rate of 10 °C/min from 28 °C to 800 °C. Dynamic mechanical properties of samples were determined by dynamic thermomechanical analysis (Netzsch DMA 242E, 1 °C/min, 1 Hz, 30 °C~220 °C, Selb, Germany). The three-point bending test was conducted on an AGS-X electronic testing machine, according to the standard GB/T9341-2008. DSC curing curves of EP and other EP nanocomposites were obtained by with a differential scanning calorimeter (DSC25, TA Instruments, New Castle, DE, USA), at the heating rate of 10 °C/min from 30 to 250 °C.

## 3. Results and Discussion

### 3.1. Synthesis and Characterization of LDH@BP Nanoparticles

**Scheme 2:**
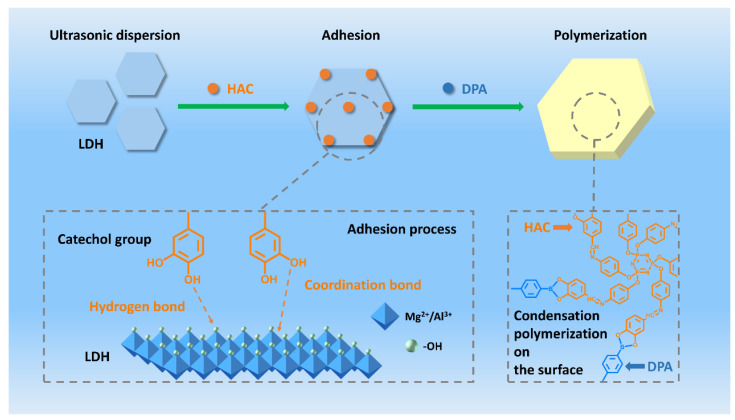
The synthesis process of LDH@BP.

The synthesis strategy of LDH@BP nanoparticles is based on the stable adsorption of catechol groups and the condensation polymerization between catechol groups and phenylboric acid groups [34]. An accurate and stable method, the synthesis strategy is shown in Scheme 2. Firstly, to introduce the phosphazene component and the DOPO component into the monomer, we synthesized multi-armed catechol monomers and two-armed phenylboric acid monomers with rich flame-retardant elements by the Schiff base reaction and Kabachnik–Fields reaction. Then, due to the adsorption of the catechol group, it can form a hydrogen bond with the hydroxyl group on the surface of the LDH, or form a coordination bond with the metal cation of LDH, so that it can be stably adsorbed on the surface of the nanosheet. Finally, based on the above, the condensation polymerization of the catechol monomer and phenylboric acid monomer is restricted to the surface of the LDH, so as to ensure the stable and efficient preparation of LDH@BP nanoparticles.

**Figure 1:**
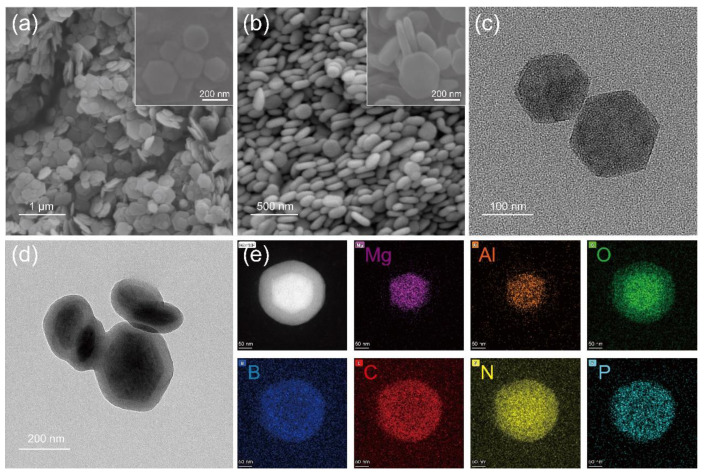
SEM images of (**a**) LDH, (**b**) LDH@BP. TEM images of (**c**) LDH, (**d**) LDH@BP. (**e**) HAADF image and corresponding EDX mappings of LDH@BP.

The morphology changes of LDH nanosheets were investigated with SEM and TEM. As shown in Figure 1a,c, the original LDH nanosheet presents a plate-like structure with regular hexagons, and its lateral size ranges from 100 to 200 nm. It is obvious that original LDH nanosheets exhibit regular morphology and a clear boundary, which is suitable for further modification as a basic material. Subsequently, the morphology of LDH nanosheets can change after the condensation polymerization of the catechol monomer and phenylboric acid monomer on its surface (Figure 1b,d). Clearly, the shape of LDH@BP still has the regular hexagonal shape, but the volume of the nanoparticle increases significantly: in particular, it becomes thicker, showing the expected core–shell structure. HADDF and EDX mapping images were used to further verify the formation of a BP shell (Figure 1e), and the associated EDX intensity spectra of LDH and LDH@BP are demonstrated in Figure S4. It can be clearly observed that Mg and Al are distributed in the core of LDH@BP, maintaining the hexagonal pattern, while the area of the presented central pattern is relatively small. On the contrary, the appearance of B, C, N, O, and P on the coated shell further confirms the successful formation of the core–shell nanoparticle.

**Figure 2:**
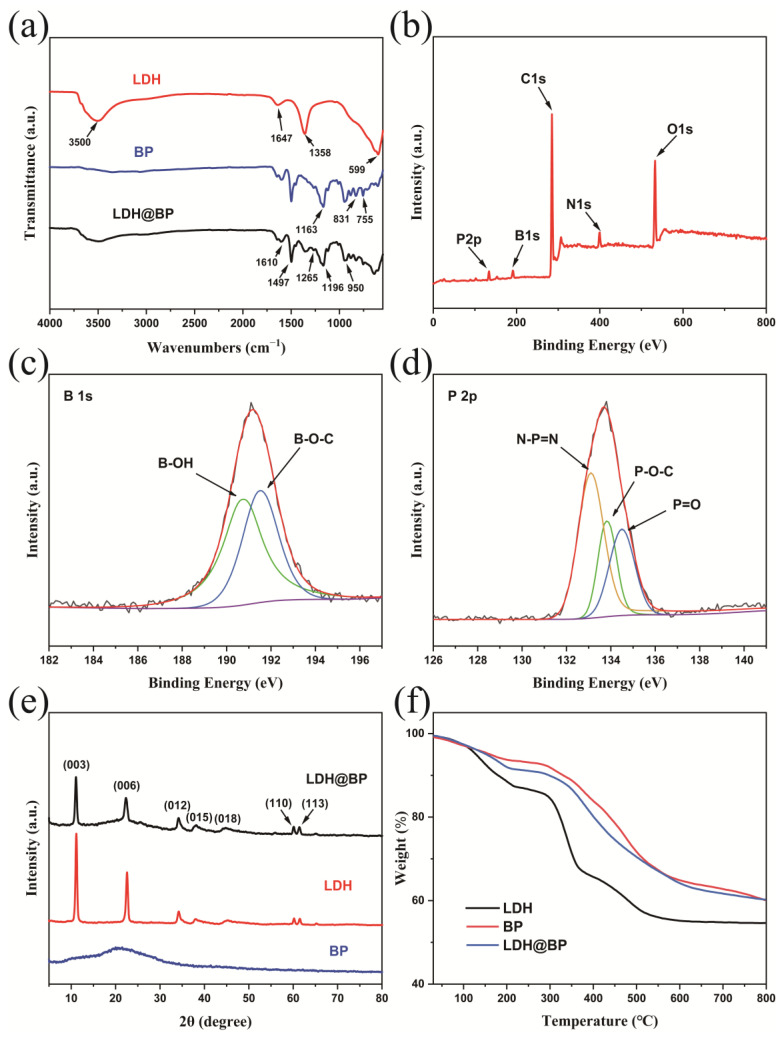
(**a**) FTIR spectra of LDH, BP and LDH@BP. (**b**) XPS full spectra of LDH@BP, and the corresponding high resolution spectra of (**c**) B 1s, (**d**) P 2p. (**e**) XRD patterns, (**f**) TGA curves of LDH, BP and LDH@BP.

The elemental composition and chemical structure of LDH, BP, and LDH@BP were studied with FTIR and XPS (Figure 2a–d). The infrared spectra of the original LDHs show typical characteristic peaks. In detail, the typical peaks of the LDH spectrum at 3500 and 1647 cm^−1^ can be ascribed to the O-H stretching vibration on the surface of the plate and the O-H bending vibration of the interlayer water molecules, respectively [35]. The absorption peak at 1358 cm^−1^ is attributed to the interlayer CO_3_^2−^ anion (derived from ambient CO_2_ during the preparation process), and the characteristic M-O stretching vibrations are observed around 599 cm^−1^ [32]. By observing the infrared spectrum of LDH@BP, it can be found that the coated nanoparticles still have all the characteristic peaks that should be attributed to LDH, which indicates that the coated LDH nanoparticles do not change the original LDH chemical structure. However, the FTIR spectra of LDH@BP and LDH also show a great difference, which is due to the successful formation of a boronate polymer shell on the surface of the LDH nanosheets. Specifically, LDH@BP has additional characteristic peaks similar to boronate polymer. For instance, characteristic peaks of boronate polymer emerged at 1610 cm^−1^ (C=N stretching vibration), 1497 and 950 cm^−1^ (benzene ring structure), 1265 and 755 cm^−1^ (corresponding to the P=O and P-O-Ph characteristic absorption of DOPO, respectively), 1196 and 831 cm^−1^ (corresponding to the P=N and P-N characteristic absorption of phosphonitrile structure, respectively), 1350 cm^−1^ (B-O-C bond; it closes to the CO_3_^2−^ anion absorption peak), and 1163 cm^−1^ (B-C stretching vibration) [34,36,37]. In addition, the element composition and chemical structure of LDH@BP were further analyzed with XPS, and Figure 2b shows the XPS full spectrum of LDH@BP. It is worth noting that because the effective detection depth of XPS is less than the thickness of the boronate polymer shell, as expected, the chemical signals associated with metallic elements involving LDHs are not recognized in the spectrum, and the actual result is to analyze the relevant chemical information of organic components for boronate polymer (this phenomenon symbolizes the high efficiency and stability of the coating program). Obviously, the signals of five elements, including C, N, O, B, and P, can be detected in the full spectrum of XPS. Furthermore, Figure 2c,d illustrate the high-resolution XPS spectra of B 1s and P 2p, respectively. The high resolution of B 1s spectra can be deconvoluted into two peaks, attributed to 190.7 eV (B-OH, produced by unreacted boric acid groups on the surface of the boronate polymer shell), and 191.5 eV (B-O-C, derived from the condensation polymerization between catechol groups and phenylboric acid groups), respectively [34]. Similarly, the high resolution of P 2p spectra can be deconvoluted into three peaks, attributed to 133.1 eV (N=P-N), 133.8 eV (P-O-C), and 134.5 eV (P=O), respectively [37], which are derived from the phosphazene component and DOPO component. In conclusion, the above results also prove the successful formation of a boronate polymer shell, which means that we have successfully introduced rich flame-retardant elements into the system. Moreover, it is rather remarkable that there exists a large number of unreacted hydroxyl groups on the surface of the boronate polymer shell (these hydroxyl groups are derived from residual catechol groups and phenylboric acid groups), which can react with EP to improve the interface interaction among LDH@BP and the EP, so as to enhance the compatibility and dispersion of nanoparticles in the EP substrate.

The crystalline structures of LDHs, BP, and LDH@BP were measured with XRD (Figure 2e). Evidently, the original LDHs have seven distinct diffraction peaks at 11.1°, 22.6°, 34.1°, 37.9°, 45.1°, 60.1°, and 61.5°, which correspond to the characteristic crystal face (003), (006), (012), (015), (018), (110), and (113) of CO_3_^2−^-LDH [20,30]. As for the BP, its spectrum exhibits only a relatively wide diffraction peak at about 20.6°, which indicates the amorphous structure of the boronate polymer. Subsequently, for the LDH@BP, it retains both the typical diffraction peak of the LDH and the amorphous diffraction peak of the boronate polymer. A further SEAD test was conducted on a single LDH@BP nanoparticle (Figure S5), and it is found that the spectrum shows a typical single crystal diffraction of LDH, which indicates that the crystal structure of LDH did not change after the boronate polymer coating process [38]. As expected, we also observe the presence of diffuse round spots in the spectrum, which is derived from the boronate polymer shell coated onto the surface of the LDH, further supporting the exact target structure. Furthermore, the thermal stability of nanoparticles was determined with TGA (in a nitrogen atmosphere), and the results are shown in Figure 2f. From the TGA curve, the thermal degradation process of LDH is divided into two obvious weight-loss stages, and the thermal degradation behavior below 300 °C is mainly ascribed to the loss of water molecules adsorbed on the surface, while the severe thermal degradation behavior between 300 °C and 600 °C is mainly attributed to the removal of water molecules and CO_3_^2−^ anions between layers and the dihydroxylation process of laminates [32]. After 600 °C, the curve enters the plateau stage with a stable mass, and the residual mass of LDH at 800 °C is 54.63%. BP has a residual mass of 60.13% at 800 °C, which demonstrates that the designed boronate polymer has excellent thermal stability. The TGA curve of LDH@BP shows that the early thermal degradation trend is the result of the co-degradation of LDH and BP. Surprisingly, compared with the theoretical co-degradation value (58.68%), the thermal stability of LDH@BP is observed to be improved, and the final residual mass of LDH@BP is 60.24% at 800 °C, which is greater than that of pure boronate polymer. This phenomenon may be related to the synergistic promotion of catalytic carbonization by LDH and BP. The coating of the boronate polymer shell is beneficial to enhancing the thermal stability of nanoparticles, which can also help to improve the flame retardancy of EP nanocomposites.

### 3.2. Dispersion and Compatibilities

**Figure 3:**
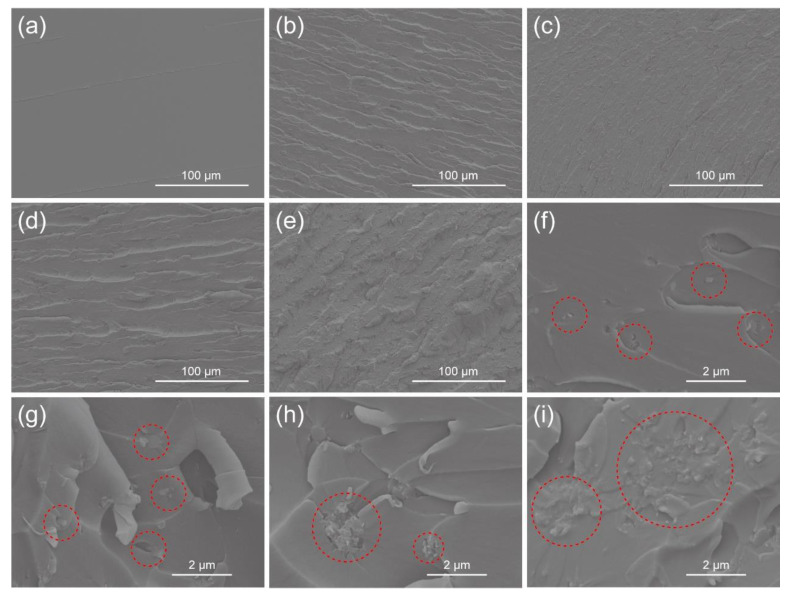
SEM images of the fracture surfaces of (**a**) EP, (**b**) EP/LDH5, (**c**) EP/LDH@BP2.5, (**d**) EP/LDH/BP5, (**e**) EP/LDH@BP5. The corresponding amplified SEM images of (**f**) EP/LDH@BP2.5, (**g**) EP/LDH@BP5, (**h**) EP/LDH5, (**i**) EP/LDH/BP5.

It is well known that the composition and structure of nanoparticles are crucial to the comprehensive properties of EP nanocomposites. Simultaneously, the dispersion of nanoparticles and the interfacial interaction between nanoparticles and the matrix are also factors which cannot be ignored, to determine the mechanical and flame-retardant properties of EP nanocomposites. To investigate the dispersion and compatibility of LDH@BP in the EP matrix, frozen fracture surfaces of EP and EP nanocomposites were characterized with SEM (Figure 3a). The fracture surface of EP shows a representative brittle fracture phenomenon with a smooth fracture surface. With the addition of nanofillers (Figure 3b–e), the fracture surface of EP composites become rough and uneven to various degrees, which manifests the conspicuous characteristics of ductile fracture. Compared with LDH, the addition of LDH@BP makes the fracture surface of EP composite grow more coarse, which indicates that the coating of the BP shell significantly promotes the interface force and enhances the compatibility between the nanoparticles and the EP matrix, so that the state of EP nanocomposites changes from brittle fracture to ductile fracture during the fracture process. Then, we further enlarged the corresponding SEM images to observe the dispersion of nanoparticles in the EP matrix and the stress state of EP nanocomposites during the fracture process (Figure 3f–i). It is evident that LDH@BPs are uniformly dispersed in the EP matrix. This is attributed to the way the coated BP shell prevents the tendency of aggregation between LDHs, which makes LDH@BP have excellent dispersion in the EP matrix. Additionally, most LDH@BPs are evenly distributed around cracks, which shows the strong interfacial interaction between LDH@BP and the EP matrix. This phenomenon demonstrates that LDH@BP can prevent continuous crack propagation during the fracture process, which gives EP nanocomposites better resistance to deformation. On the contrary, in control group, with the increased loading of LDH (or the control group of the physical blending of LDH and BP nanospheres), we can observe a serious agglomeration phenomenon of nanoparticles in the matrix. Certainly, the above aggregation phenomena are attributed to the strong van der Waals force and the hydrophilicity of unmodified LDHs, which result in a large number of LDHs being stacked in the EP matrix and ultimately lead to the poor dispersion of the nanosheets.

**Figure 4:**
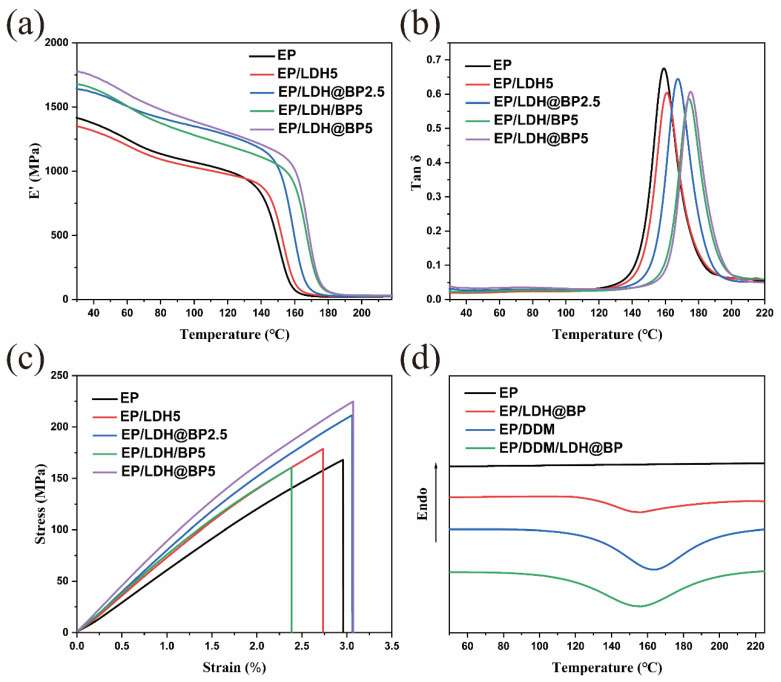
(**a**) Storage modulus curves and (**b**) tan δ curves of EP and other EP nanocomposites with DMA, (**c**) the stress–strain curves of EP and other EP nanocomposites with the three-point bending tests, (**d**) the DSC curing curves of EP, EP/LDH@BP, EP/DDM and EP/DDM/LDH@BP.

In principle, the good dispersion of LDH@BPs in the EP matrix can support the enhancement of the mechanical properties of EP nanocomposites. Therefore, we investigated the mechanical properties of EP and other EP nanocomposites with DMA and three-point bending tests. The related image results obtained are shown in Figure 4a–c, and the corresponding parameter results are demonstrated in Table 1. As expected, contrasted with EP, the mechanical properties of EP/LDH@BP are gradually improved with the increased loading of LDH@BP. In particular, the storage modulus, glass-transition temperature, flexural modulus, and flexural strength of EP/LDH@BP5 are 1774.9 MPa, 175.2 °C, 3049.8 MPa, and 105.6 MPa, respectively, and compared with EP, the above corresponding figures increased by 25.5%, 10.1%, 37.8%, and 19.1%, respectively.

In addition, previous studies have demonstrated that the introduction of unmodified MgAl-LDH nanosheets can hinder the curing reaction of EP and negatively affect the curing status of the entire system [39]. Therefore, to comprehensively appreciate the chemical properties and distribution state of modified LDH@BP nanoparticles in the EP matrix, we measured the curing curves with DSC to investigate the curing behavior among different control groups (Figure 4d). Evidently, there is no exothermic peak corresponding to the curing reaction in pure EP without the addition of curing agent; as envisaged, the EP/LDH@BP mixture shows a significant exothermic peak after the introduction of LDH@BP, which means that the coated LDH@BP nanoparticles can participate in the curing process of EP. The phenomenon benefits from active groups on the boronate polymer shell, such as unreacted residual hydroxyl groups, or tertiary amines on the DPA [40]. Under the influence of LDH@BP, the curing temperature of EP/DDM/LDH@BP shifts to a lower temperature than that of the traditional EP/DDM curing system, symbolizing that the introduction of LDH@BP can catalyze the curing reaction of EP and accelerate the crosslinking process between the system. Combined with previous experimental phenomena and theoretical judgment, we speculate that such experimental results are related to the following factors. First, the coated boronate polymer shell effectively restricts the aggregation of LDHs, thus allowing LDH@BPs to be evenly dispersed in the EP matrix. Secondly, the residual reaction groups on the boronate polymer shell can take part in the curing process of epoxy resin, which improves the crosslinking state of EP/LDH@BP. Finally, contrasted with unmodified LDH, LDH@BP has better compatibility and interfacial interaction with the EP matrix, enabling the rigid nanoparticles to be properly embedded into the matrix, which can promote the transfer of load when the matrix is under stress, and thus improve the mechanical properties of EP/LDH@BP. On the contrary, due to the severe problems of dispersion and discordant interfacial interaction, the mechanical properties of EP/LDH have not been significantly improved with the addition of unmodified LDH, and some parameters, such as the energy storage modulus and elongation at break, have even decreased.

### 3.3. Thermal Stability

**Figure 5:**
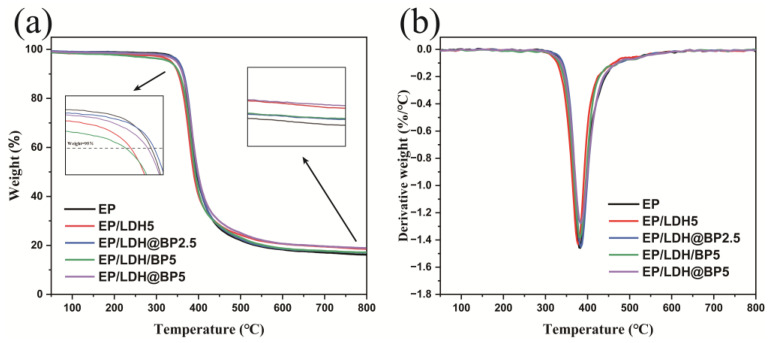
(**a**) TGA curves and (**b**) DTG curves of EP and other EP nanocomposites under N_2_ atmosphere.

The thermal stability of EP and other EP nanocomposites was characterized with TGA in a nitrogen atmosphere. The results are shown in the TGA and DTG curves (Figure 5), and the corresponding detailed parameters are listed in Table 2. As can be seen from the fluctuation of TGA curves, all samples exhibit a major degradation process, which is ascribed to the degradation of EP polymer macromolecular chains. T_5%_ is defined as the initial pyrolysis temperature; owing to the early endothermic decomposition of LDH and BP, the T_5%_ of EP/LDH5 and EP/LDH/BP5 are significantly lower than that of pure EP. Compared with other control groups, the T_5%_ of EP/LDH@BP shows a higher value, consistent with the previous experimental phenomenon (TGA result of LDH@BP), which is mainly because the BP shell delays the thermal degradation process of LDH. The addition of LDH@BP does not cause significant fluctuation of T_50%_ and T_max_ of EP nanocomposites, but the maximum mass loss rate of EP/LDH@BP decreases gradually with the increased loading of LDH@BP. It can be found that by observing the residual mass of the sample at 800 °C, compared with the residual yield of EP (16.2%), the residual yield of EP nanocomposite gradually advances with the increased content of nanoparticles. Moreover, contrasting with EP/LDH/BP, EP/LDH@BP5 shows the highest residual mass (18.9%). On the one hand, it can be ascribed to the excellent dispersion of LDH@BPs in the EP matrix, which suppresses the movement of macromolecular chains of EP nanocomposites and delays the process and magnitude of degradation. On the other hand, this may be owing to the synergistic catalytic carbonization effect between LDH and BP, which is closely related to the bimetallic oxide barrier layer formed by LDH at high temperature, and the formation of a dense char layer promoted by the decomposition of the phosphorous component of BP. The above results illustrate that EP/LDH@BP nanocomposites have enhanced thermal stability and catalytic carbonization performance, which are helpful to analyze and comprehend the flame-retardant mechanism of EP/LDH@BP.

### 3.4. Flame Retardancy of EP Nanocomposites

**Figure 6:**
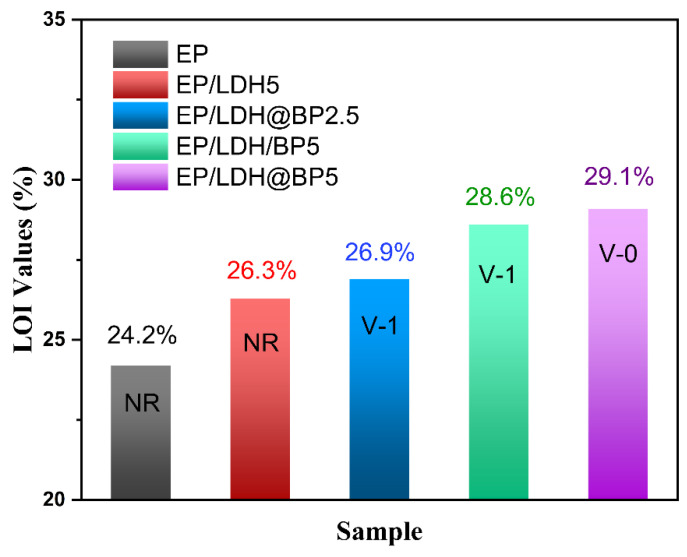
LOI and UL-94 image results of EP, EP/LDH5, EP/LDH@BP2.5, EP/LDH/BP5, and EP/LDH@BP5 nanocomposites.

The fire safety property of EP is very crucial to protect people’s life and property. First, the flame retardancy of EP and other EP nanocomposites was investigated by using LOI and UL-94 vertical burning tests (Figure 6), and the detailed parameters obtained from these tests are demonstrated in Table S1. As expected, the LOI of pure EP is only 24.2%, and it manifests no rating in the UL-94 vertical burning tests; there exists severe dropping during the combustion process, indicating that epoxy resin without flame-retardant additives is a combustible material. Subsequently, we introduced several distinct nanoparticles with different contents and structures into EP and observed the flame retardancy of EP/LDH, EP/LDH/BP and EP/LDH@BP nanocomposites. Apparently, following the raised loading of LDH@BP, the flame retardancy of EP/LDH@BP nanocomposites has been greatly improved. When 2.5 wt% and 5 wt% LDH@BP are added into EP, the LOI of EP/LDH@BP can reach 26.9% and 29.1%, respectively. Meanwhile, the phenomenon of melting drops in the combustion process can be effectively inhibited. It is worth noting that the EP nanocomposites achieve a rating of V-0 in the vertical burning tests when the content of LDH@BP reaches 5 wt%, which means that the test splines are extinguished within a short time after ignition. As for the control group, the LOI of EP/LDH5 is only 26.3%, and it is not rated in the UL-94 tests. The LOI of the EP/LDH/BP5 is only 28.6%, and the UL-94 test rating reduces to V-1, which is obtained by adding 5 wt% of physically blended LDH and BP microspheres to the EP matrix. The above results indicate that the modification of LDHs by BP and the improved dispersion of nanoparticles can provide EP nanocomposites a better ability to resist combustion.

**Figure 7:**
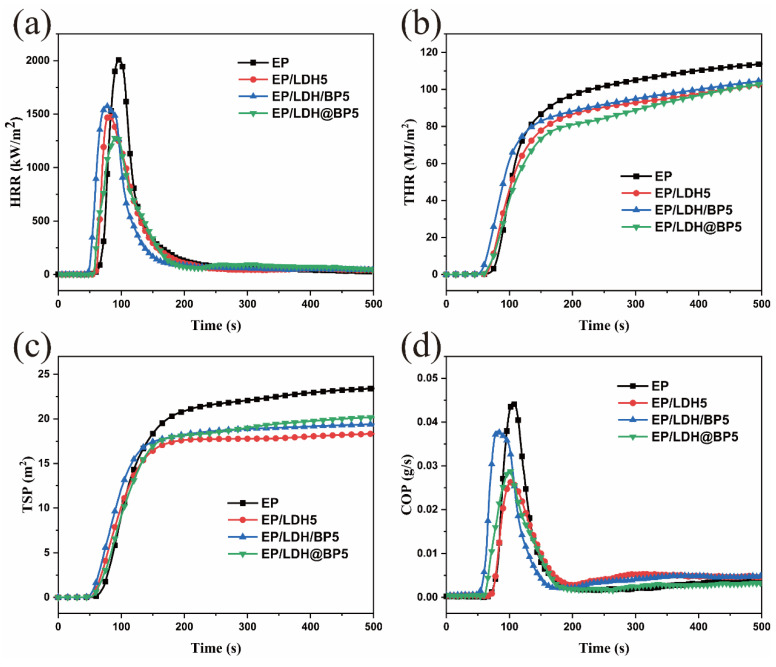
(**a**) HRR, (**b**) THR, (**c**) TSP, and (**d**) COP curves of EP and other EP nanocomposites.

To evaluate the rate of heat release and smoke generation of EP, EP/LDH, EP/LDH/BP, and EP/LDH@BP nanocomposites during intense combustion, the flame retardancy of EP nanocomposites containing 5 wt% nanoparticles was further investigated by cone calorimeter tests. The resulting HRR, THR, TSP, and COP curves are shown in Figure 7, while the corresponding parameter results are demonstrated in Table 3. Pure EP exhibits high PHRR, THR, and TSP values, which are 2010 kW/m^2^, 113.7 MJ/m^2^, and 23.41 m^2^, respectively. When 5 wt% LDH, LDH/BP, and LDH@BP are added to EP, the PHRR of EP nanocomposites decrease by 25.9%, 21.7% and 35.9%, respectively. It can be surmised that the PHRR of EP/LDH@BP5 decreased considerably, which can be mainly by reason of the physical barrier effect from the LDH and the boronate polymer shell containing rich flame-retardant elements, which can form an efficient and dense protective char layer during combustion. For the PHRR values, it is a remarkable fact that the incorporation of LDH@BP can improve the fire resistance of EP nanocomposites more effectively than the addition of a simple physical blend of LDH and BP. When t = 500 s, the THR declines of EP/LDH5, EP/LDH/BP5, and EP/LDH@BP5 are similar, and their THR values are 102.7, 104.7, and 102.9 MJ/m^2^, respectively. The rate of smoke release during combustion is also an important indicator to evaluate the fire resistance of EP/LDH@BP. As exhibited in Figure 7c,d, the incorporation of LDH@BP effectively reduces the TSP of EP nanocomposites. Especially for COP, LDH@BP nanocomposites efficiently inhibit the total amount of carbon monoxide produced by EP during the complete combustion process, greatly reducing the toxicity of the smoke. Overall, based on the results of the LOI, UL-94 tests, and cone calorimeter tests above, EP/LDH@BP5 manifests the most efficient flame retardancy in different control groups.

### 3.5. Flame-Retardant Mechanism

**Figure 8:**
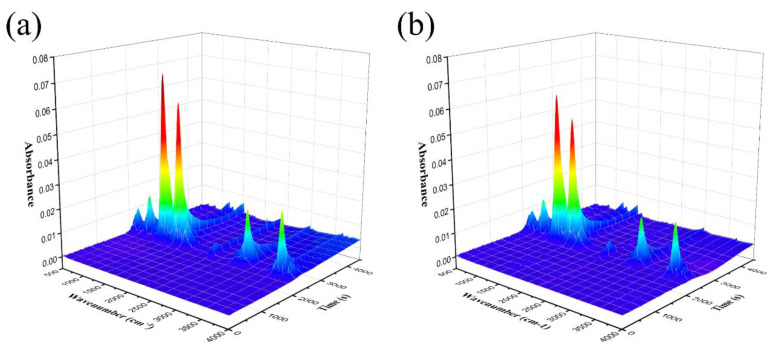
3D TG-FTIR spectra of pyrolysis product releases for (**a**) EP and (**b**) EP/LDH@BP5.

**Figure 9:**
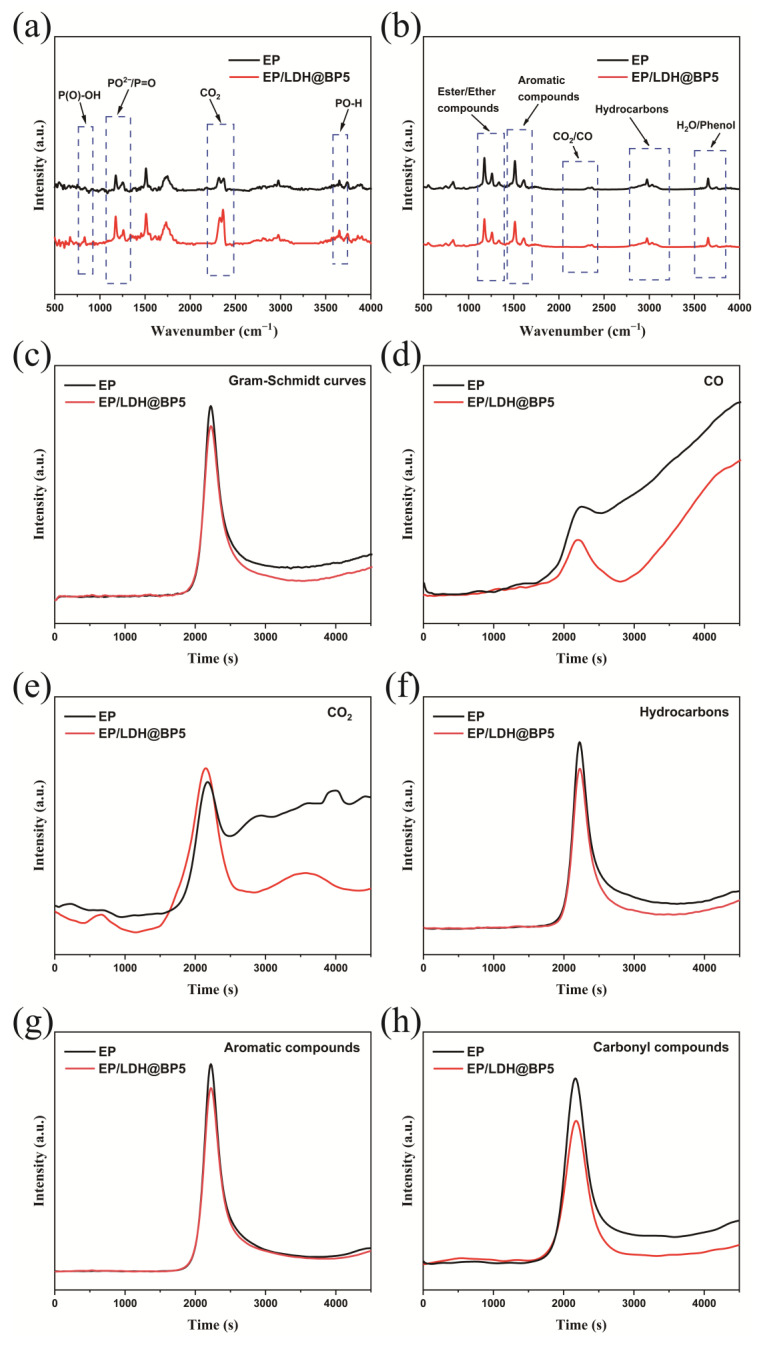
FTIR spectra of pyrolysis product releases for EP and EP/LDH@BP5 at the initial decomposition temperature (**a**) and the maximum decomposition temperature (**b**). Absorbance of pyrolysis products for EP and EP/LDH@BP5: (**c**) Gram–Schmidt curves, (**d**) CO, (**e**) CO_2_, (**f**) hydrocarbons, (**g**) aromatic compounds, (**h**) carbonyl compounds.

To further inquire into the flame-retardant mechanism of LDH@BP, TG-FTIR was applied to characterize the exhaust-gas elimination mechanism of EP/LDH@BP5 during combustion. The 3D TG-FTIR spectra of the pyrolysis product releases of EP and EP/LDH@BP5 are demonstrated in Figure 8. The variation trend of volatile products of EP and EP/LDH@BP5 in the gradual combustion course is similar, but the absorbance of EP/LDH@BP5 in the spectrum is lower than that of EP. Figure 9a provides the FTIR spectra of pyrolysis product releases of EP and EP/LDH@BP5 at the initial decomposition temperature. Compared with EP, EP/LDH@BP5 shows higher characteristic peaks at 826, 1174, 1256, and 3652 cm^−1^, associated with P(O) -OH, PO^2−^, P=O, and PO-H, respectively; they are produced by compounds containing phosphorus in the gas phase. Meanwhile, at the initial degradation temperature, compared with EP, the absorbance of carbon dioxide for EP/LDH@BP5 displays higher intensity, which may be attributed to the release of interlayer anion CO_3_^2−^ by the early thermal degradation of LDH, and the presence of boronate polymer can also catalyze the complete conversion of the oxide. In addition, there were no boron-containing compounds observed in the pyrolysis products. Observing the FTIR spectra of pyrolysis product releases at the maximum decomposition temperature (Figure 9b), it is apparent that the pyrolysis products produced by both at the maximum decomposition temperature are almost identical. The main gaseous products can be summarized into several substances, for instance, ester/ether compounds (1175/1258 cm^−1^), aromatic compounds (1450–1650 cm^−1^), CO/CO_2_ (2150/2357 cm^−1^), hydrocarbons (2800–3100 cm^−1^), and water/phenol (3500–4000 cm^−1^) [41,42]. These results certify that the addition of LDH@BP does not influence the main gaseous pyrolysis products of EP during combustion. Subsequently, for further systematic comparison, the intensity curves of total pyrolysis gaseous products of EP and EP/LDH@BP5 over time are shown in Figure 9c, and Figure 9d–h, respectively, demonstrating the intensity variation trend of several representative pyrolysis products in the combustion process. The Gram–Schmidt curves demonstrate that the introduction of LDH@BP reduces the release of gaseous pyrolysis products from EP nanocomposites. In particular, LDH@BP can obstruct the release of flammable and toxic smoke such as aromatic compounds, carbon monoxide, hydrocarbons, and carbonyl compounds, and significantly reduce the harm to the human body from toxic substances that are produced by EP during intense combustion. This smog-suppressing function is attributed to the synergistic action of LDH and the boronate polymer shell. First of all, LDH has the physical barrier effect and the adsorption properties of gaseous pyrolysis products. Secondly, the organic component of the boronate polymer shell can promote the well-distributed dispersion of LDH in the EP matrix and catalyze the complete degradation of EP/LDH@BP5 during intense combustion from the perspective of the condensed phase, which advances the formation of compact char layers on the surface of EP/LDH@BP nanocomposites, thus inhibiting the volatilization of gas phase pyrolysis products.

**Figure 10:**
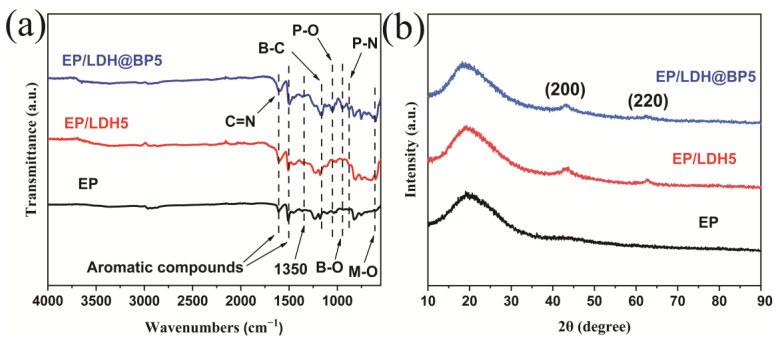
(**a**) FTIR spectra, (**b**) XRD patterns of char residues of EP and other EP nanocomposites.

The chemical structure and composition of the char residues of EP and other EP nanocomposites after combustion were investigated with FTIR and XRD (Figure 10). As shown in Figure 10a, the infrared spectra of EP, EP/LDH5, and EP/LDH@BP5 also show several identical strong absorption peaks from aromatic compounds (1608 and 1500 cm^−1^; C=N bond is also contained within the peak of 1608 cm^−1^) [43]. Different from pure EP, EP/LDH5 and EP/LDH@BP5 have a wide absorption peak at 550–750 cm^−1^, which is ascribed to M-O vibration, indicating that the char residues in both of them contain bimetallic oxides, which were formed by LDHs at high temperature [44]. Furthermore, for the char residues of EP/LDH@BP5, the weak absorption peak at 1350 cm^−1^ implies the breakdown of B-O-C bonds and the release of interlayer anions in the thermal decomposition circumstance. Several additional strong absorption peaks are assigned to the boron–oxygen network B-O vibration (949 cm^−1^), B-C bond (1165 cm^−1^), P-O bond (1064 cm^−1^), and P-N bond (880 cm^−1^), indicating that the boron-containing component of the boronate polymer tends to interact with carbon or form boron oxygen compounds and remain in the condensed phase at a high temperature [45]; additionally, the phosphate-containing component can form phosphoric acid and crosslinked phosphorus oxynitride during the pyrolysis process [41]. They are able to promote the construction of high quality and complete char layers. The XRD patterns of char residues from EP, EP/LDH5, and EP/LDH@BP5 are shown in Figure 10b. All of the XRD patterns also exhibit a wide diffraction peak at about 20°, which corresponds to (002) diffraction of graphite. As for the XRD patterns of EP/LDH5 and EP/LDH@BP5 residual chars, there are two additional characteristic peaks at 43.3° and 63.2°, which are ascribed to the (200) and (220) crystal face of the MgAlO phase, respectively [46]. These extra peaks result from the decomposition of LDH at high temperature, and this phenomenon is consistent with the previous characterization results of FTIR spectra.

**Figure 11:**
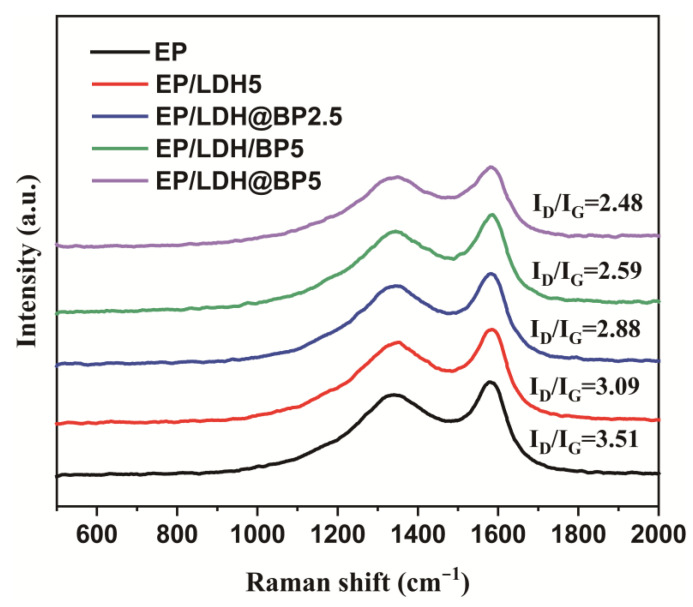
Raman spectra of char residues of EP and other EP nanocomposites.

Raman spectroscopy was used to investigate the extent of graphitization of the char residues from EP and other EP nanocomposites after intense combustion (Figure 11). Apparently, two peaks can be observed for each Raman spectrum in the figure; these are peak D at 1350 cm^−1^ and peak G at 1580 cm^−1^. It is well known that the intensity ratio between peak D and peak G, namely I_D_/I_G_, is commonly utilized to evaluate the extent of graphitization of residual chars: the smaller the value of I_D_/I_G_, the higher the degree of graphitization of the tested substance. By calculating the I_D_/I_G_ ratio of EP and other EP nanocomposites in the spectra, the acquired corresponding values of EP, EP/LDH5, EP/LDH@BP2.5, EP/LDH/BP5, and EP/LDH@BP5 are 3.51, 3.09, 2.88, 2.59, and 2.48, respectively. It can be observed exactly that the I_D_/I_G_ value of EP/LDH@BP gradually decreases with the increment of LDH@BP content, and the decrease amplitude is higher than that of EP/LDH5 or EP/LDH/BP5. This demonstrates that EP/LDH@BP can advance the formation of a more continuous and dense char layer after combustion, which avoids the sustaining transfer of flammable gas and heat from the outside, thus enhancing the flame-retardant property of EP/LDH@BP5 nanocomposites.

**Figure 12:**
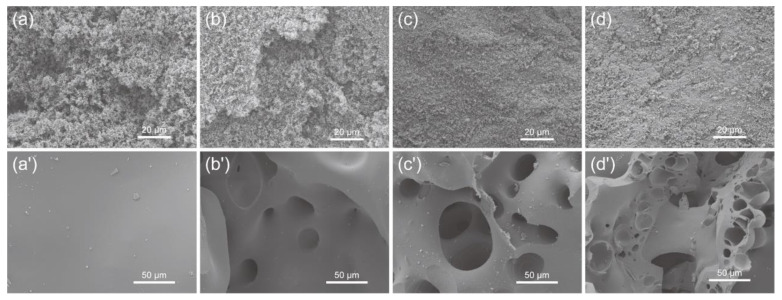
SEM images of the external char residues from (**a**) EP, (**b**) EP/LDH5, (**c**) EP/LDH/BP5 and (**d**) EP/LDH@BP5; SEM images of the inner char residues from (**a′**) EP, (**b′**) EP/LDH5, (**c′**) EP/LDH/BP5 and (**d′**) EP/LDH@BP5.

The residual char morphologies of EP, EP/LDH5, EP/LDH/EP5, and EP/LDH@BP5 were further characterized with SEM (Figure 12). The SEM images of the external char residues are shown in Figure 12a–d. The external char residues of EP present loose characteristics, and there exists a large number of gaps. It is difficult to effectively block the passage of combustible gas and heat with this char layer structure when EP is ignited. For the EP/LDH5, with the addition of LDHs, it can be confirmed that the feature of external char residues becomes cohesive and more compact than before. However, because of the agglomeration of LDHs in the matrix, the char layer still shows discontinuous characteristics. On the contrary, as expected, the addition of LDH@BP enables the EP/LDH@BP5 nanocomposites to form continuous and dense external char residues during the combustion procedure, which can more efficiently act as a solid barrier to promote the fire resistance of EP/LDH@BP5. Additionally, the morphologies of the inner char residues between EP and other EP nanocomposites also display a great difference (Figure 12a′–d′). Compared with EP, which has a smooth surface with fewer pores, the inner char residues of other EP nanocomposites exhibit a more porous structure with the addition of nanoparticles: EP/LDH@BP5, especially, has the most uniform and dense pores. The large number of small pores is caused by the non-flammable gas released by LDHs and BP during the intense combustion of EP nanocomposites, which derives from water vapor and carbon dioxide released by LDHs at high temperatures (from interlayer water molecules and CO3^2−^ anion), and non-flammable gas produced by the combustion process of boronate polymer.

**Figure 13:**
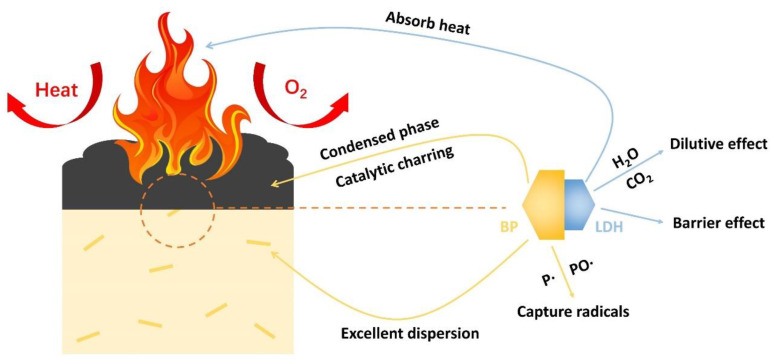
Schematic illustration of the flame retardancy mechanism of LDH@BP.

Combined with the above analysis involving the gas phase and the condensed phase, a mechanism for LDH@BP’s enhancing the flame retardancy of EP/LDH@BP nanocomposites was proposed (Figure 13). The excellent flame retardancy of EP/LDH@BP nanocomposites is the result of synergistic action of LDH and the boronate polymer shell. First of all, the outstanding dispersion and compatibility of nanoparticles in the EP matrix is the necessary condition to improve the flame retardancy of EP/LDH@BP nanocomposites; the boronate polymer shell effectively limits the tendency of mutual accumulation between LDH nanosheets, so that LDH@BP can be evenly distributed in the matrix, and the remaining reaction groups on the surface of the boronate polymer can interact with the macromolecular chains of EP, which efficiently participate in the curing crosslinking procedure of EP and improve the interfacial interaction between LDH@BP nanoparticles and the EP matrix. Secondly, the boronate structure, phosphazene component, and DOPO component, which are contained in BP, can act together in different phases during the combustion process of EP. In the gas phase, P-containing free radicals can be generated to block the chain reaction of sustaining combustion; in the condensed phase, the boron-containing component, phosphoric acid, and crosslinked phosphorus oxynitride can promote the formation of continuous and dense protective char layers to insulate the heat and fuel from the outside. Finally, in the thermal decomposition process, the LDH nanosheets, as the core component, absorb heat, release interlayer H_2_O and CO_2_ molecules to dilute the combustible gas, and form bimetallic oxides to act as steady physical barriers to prevent heat release and smoke production during the combustion of EP/LDH@BP. Through the synergistic action of the above aspects, the flame retardancy of EP/LDH@BP nanocomposites has been significantly enhanced.

## 4. Conclusions

In this work, organic–inorganic nano-hybrid flame-retardant LDH@BP are designed, which is achieved by modifying LDH with a boronate polymer containing rich flame-retardant elements (P, N, and B). Then, we investigate the morphology and chemical structure of LDH@BP with SEM, TEM, FTIR, XPS, XRD, and other characterization methods, and successfully prove the synthesis of LDH@BP nanoparticles, while introducing them into EP to obtain EP/LDH@BP nanocomposites. As controls, EP, EP/LDH, and EP/LDH/BP nanocomposites are also prepared to observe the differences between their target properties. The results demonstrate that EP/LDH@BP exhibits the best flame retardancy and mechanical properties. With only 5 wt% LDH@BP, EP/LDH@BP5 possesses a high LOI and acquires the rating of V-0 in the UL-94 test. Compared with the other controls, EP/LDH@BP shows lower PHRR, THR, TSP, and COP values, and the toxic gases and other gaseous products volatized by EP/LDH@BP during combustion are significantly restrained. In addition, the introduction of LDH@BP improves the resistance of the nanocomposites to deformation, which benefits from the outstanding dispersion and compatibility of LDH@BP nanoparticles in the EP matrix. Based on the above facts and the detailed analysis of the gas phase and condensed phase, we propose a flame-retardant mechanism for LDH@BP to improve the fire safety of EP/LDH@BP, and hold the opinion that the excellent flame retardancy of EP/LDH@BP is the result of synergistic action by LDH and the boronate polymer. In conclusion, via a rational and stable system design, we regulate the interface interaction between LDH and the EP matrix and improve its functionality. This work thus provides a new perspective on using LDH to prepare efficient functional EP nanocomposites.

## Figures and Tables

**Table 1:** DMA and three-point bending test analysis data of EP and other EP nanocomposites.

	DMA			The 3-Point Bending Test	
Samples	T_g_ (°C)	E’ (MPa)	tan δ	Flexural Strength (MPa)	Flexural Modulus (MPa)
EP	159.0	1414.4	0.675	88.7 ± 4.0	2213.3 ± 44.1
EP/LDH5	160.8	1350.8	0.604	90.2 ± 0.9	2528.5 ± 67.6
EP/LDH@BP2.5	167.7	1639.9	0.645	102.9 ± 3.4	2687.2 ± 100.4
EP/LDH/BP5	174.5	1676.5	0.586	88.0 ± 1.8	2990.9 ± 255.7
EP/LDH@BP5	175.2	1774.9	0.607	105.6 ± 1.2	3049.8 ± 167.9

**Table 2:** TGA results of EP, EP/LDH5, EP/LDH@BP2.5, EP/LDH/BP5, and EP/LDH@BP5 nanocomposites in N_2_ atmosphere.

Samples	T_5%_/°C	T_50%_/°C	T_max_/°C	Char Yield /wt% (800 °C)
EP	350.3	395.2	382.7	16.2
EP/LDH5	335.1	387.8	377.5	18.6
EP/LDH@BP2.5	352.5	397.2	384.1	17.0
EP/LDH/BP5	330.7	391.1	382.9	17.2
EP/LDH@BP5	347.4	398.5	382.2	18.9

**Table 3:** Cone calorimeter test data for EP and other EP nanocomposites.

Sample	PHRR	THR	SPR	TSP	COP
	(kW/m^2^)	(MJ/m^2^)	(m^2^/s)	(m^2^)	(g/s)
EP	2010	113.7	0.370	23.41	0.0445
EP/LDH5	1490	102.7	0.283	18.33	0.0262
EP/LDH/BP5	1575	104.7	0.321	19.40	0.0379
EP/LDH@BP5	1288	102.9	0.282	20.17	0.0290

## Data Availability

The data supporting the findings described in this manuscript are available from the corresponding authors upon request.

## Supplementary Materials

The following supporting information can be downloaded at: https://www.mdpi.com/article/10.3390/polym15092198/s1. References [47,48,49] are cited in supplementary material. Figure S1. Synthetic procedure for HNCP, HACP, and HAC; Figure S2. Synthetic procedure for DPA; Figure S3. ^1^H (a) and ^31^P (b) NMR spectra of DPA, solvent: DMSP-d6; Figure S4. EDX spectrum of LDH and LDH@BP; Figure S5. SEAD pattern of LDH@BP; Figure S6. Digital photos of UL-94 vertical burning tests for EP, EP/LDH5, EP/LDH@BP2.5, EP/LDH/BP5, and EP/LDH@BP5 nanocomposites; Table S1. The formulation of EP and other EP nanocomposites, and the corresponding results of LOI and UL-94.
