# Supplementary material for: LDH@Boronate Polymer Core–Shell Nanoparticles: Nanostructure Design for Synergistically Enhancing the Flame Retardancy of Epoxy Resin
Source: Polymers (Basel). 2023 May 5;15(9):2198. doi: 10.3390/polym15092198 (PMC10181304; doi:10.3390/polym15092198)
Supplement: Supplementary file 1 [file polymers-15-02198-s001.zip › polymers-2359674-supplementary.pdf]

Supporting information

S1. Experimental Section

S1.1 Materials

Magnesium nitrate hexahydrate [$Mg(NO_3)_2 \cdot 6H_2O$], aluminum nitrate nonahydrate [$Al(NO_3)_3 \cdot 9H_2O$], sodium hydroxide, hexachlorocyclotriphosphazene (HCCP), p-nitrophenol, K_2CO_3, Pd/C, hydrazine hydrate (80 wt.%), 3,4-Dihydroxybenzaldehyde, 4,4'-Diaminodiphenyl ether, 4-Formylphenylboronic acid, 9, 10-dihydro-9-oxa-10-phosphaphenanthrene-10-oxide (DOPO), and 4,4'-diaminodiphenylmethane (DDM) were obtained from Aladdin Company. Diglycidyl ether of bisphenol A epoxy resin (DGEBA(E-51)) was obtained from Jinan Qingtian Chemical Co. Ltd. All reagents were used as received, without further purification.

S1.2 Synthesis of Catechol monomer HAC

HNCP, HACP, and catechol monomer HAC was synthesized according to our previous work [1]. The synthetic procedures are shown in Figure S1.

S1.3 Synthesis of hexakis-(4-aminophenoxy)-cyclotriphosphazene (HNCP)

An amount of 5.21 g HCCP was completely dissolved in 100 mL of acetone and then transferred to a three-necked flask containing 10 g of K_2CO_3 in a nitrogen atmosphere at 45℃. An amount of 12.52 g 4-nitrophenol was completely dissolved in 20 mL acetone, slowly added to the system, and reacted for 12 h. After the reaction, the precipitation was filtered and washed with acetone, methanol, and deionized water. Finally, the white powder HNCP was obtained by vacuum-drying at 60 °C. Yield: 85%. ^1H NMR (DMSO-d_6, ppm): δ=8.15~8.17 (m, 12H), 7.30~7.32 (m, 12H). ^{31}P NMR (DMSO-d_6, ppm): δ=7.28 (s, 1P).

S1.4 Synthesis of hexakis-(4-aminophenoxy)-cyclotriphosphazene (HACP)

Amounts of 4.82 g HNCP and 0.5 g Pd/C were introduced into a three-neck flask containing 100 mL anhydrous ethanol in a nitrogen atmosphere and heated to 80 °C. The mixed solution of 20mL hydrazine hydrate and 20 mL anhydrous ethanol was slowly added to the system and reacted for 24 h. After the reaction, Pd/C was removed by filtration, and the crude product was separated from the liquid reaction mixture by water precipitation. The white powder HACP was finally obtained after freeze-drying. Yield: 75%. ^1H NMR (DMSO-d_6, ppm): δ=6.50~6.52 (m, 12H), 6.41~6.44 (m, 12H), 4.91 (s, 12H). ^{31}P NMR (DMSO-d_6, ppm): δ=10.01(s, 1P).

S1.5 Synthesis of HAC

An amount of 0.392 g HACP and 0.621 g 3,4-dihydroxybenzaldehyde were completely dispersed in 40mL absolute methanol. The reaction mixture was stirred in a nitrogen atmosphere at 60 °C for 24 h. Then, the precipitate was collected by filtration, washing with absolute methanol, and drying in a vacuum. Yield: 90%. ^1H NMR (DMSO-d6, ppm): δ=8.99~10.12 (m, 12H), 7.25~7.40 (m, 12H), 7.05~7.14 (m, 12H), 6.83~6.93 (m, 12H), 6.60~6.72 (m, 6H). ^{31}P NMR (DMSO-d6, ppm): δ=9.09 (s, 1P).

S1.6 Synthesis of phenylboric acid monomer DPA

An amount of 0.4 g 4,4'-Diaminodiphenyl ether and 0.6 g 4-Formylphenylboronic acid were completely dissolved in 60 mL mixed solution of DMF and absolute methanol; the ratio of DMF to absolute methanol was 1:3. The reaction solution was stirred at room temperature and reacted for 12 hours. Then, 0.864 g DOPO was completely dissolved in 40 mL absolute methanol and slowly added to the system and reacted for 12 h. After the reaction, the crude product was separated from the liquid reaction mixture by water precipitation, washing with absolute methanol, and drying in a vacuum. The white powder

DPA was finally obtained after freeze-drying. The synthetic procedure and the corresponding NMR spectra are listed in Figure S2 and Figure S3, respectively. Yield: 85%. ^{1}H NMR (DMSO-d6, ppm): δ=8.09~8.26 (m, 4H), 6.02~8.06 (m, 34H), 4.94~5.56 (m, 2H). ^{31}P NMR (DMSO-d6, ppm): δ=28.48, 31.02 (s, 1P).

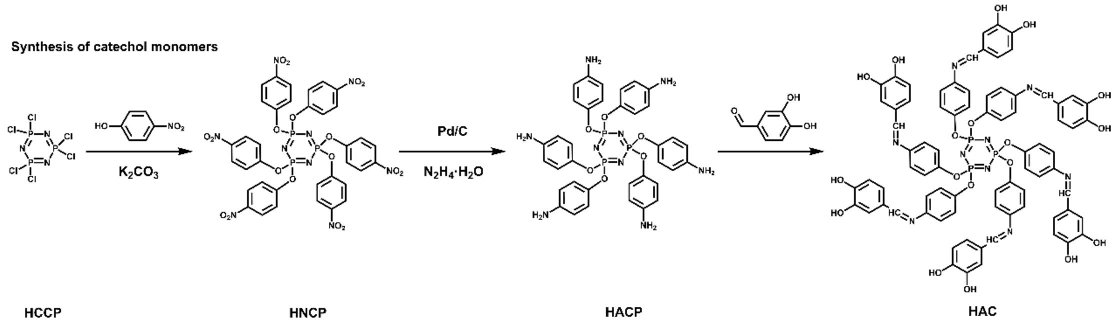

Figure S1. Synthetic procedure for HNCP, HACP, and HAC.

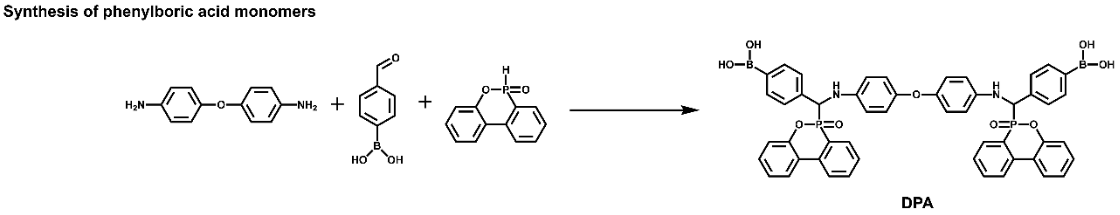

Figure S2. Synthetic procedure for DPA.

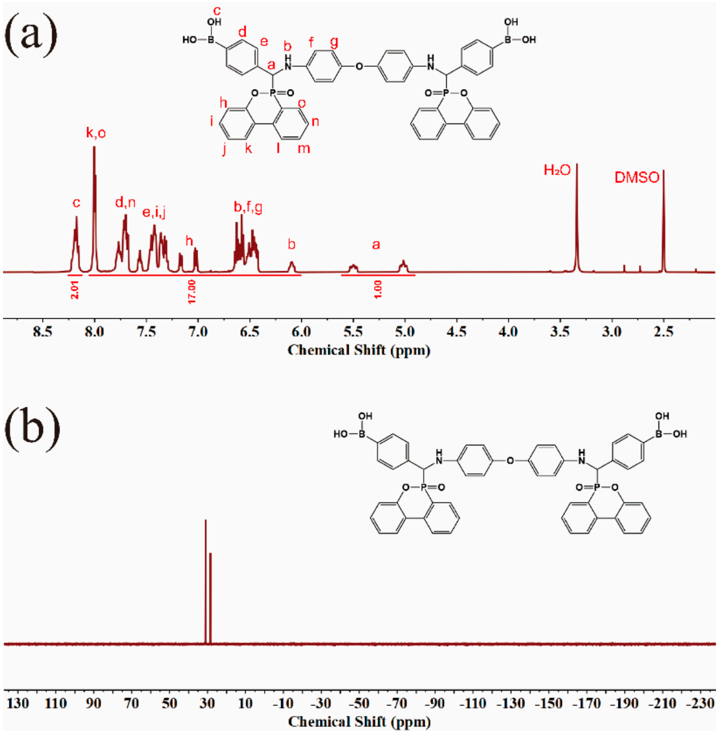

Figure S3. ^{1}H (a) and ^{31}P (b) NMR spectra of DPA, solvent: DMSP-d6.

It should be noted that due to the steric hindrance of phosphaphenanthrene groups in DPA compounds, there are two different phosphorus chemical environments in the 31PNMR spectrum of DPA, thus resulting in two chemical shifts [2,3].

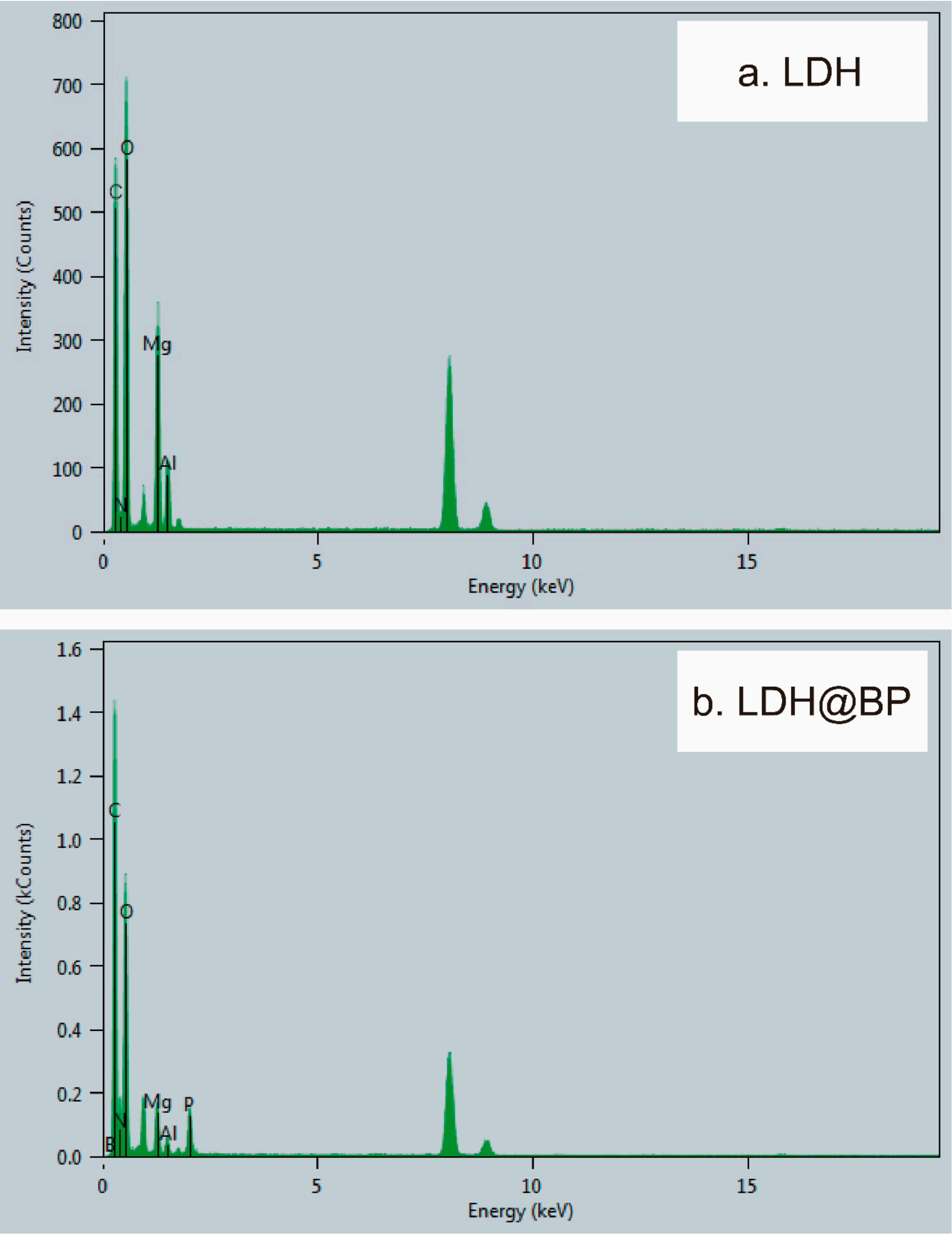

Figure S4. EDX spectrum of LDH and LDH@BP.

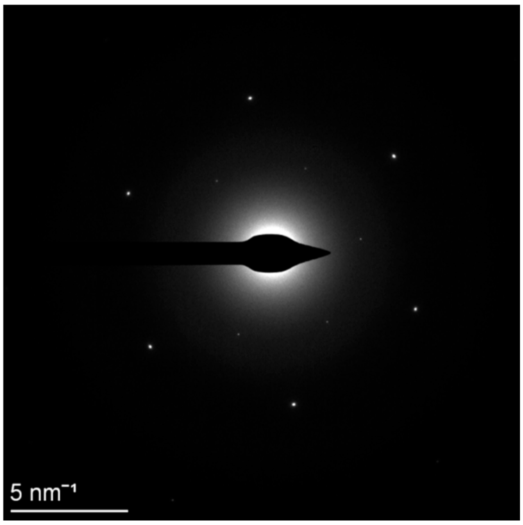

Figure S5. SEAD pattern of LDH@BP.

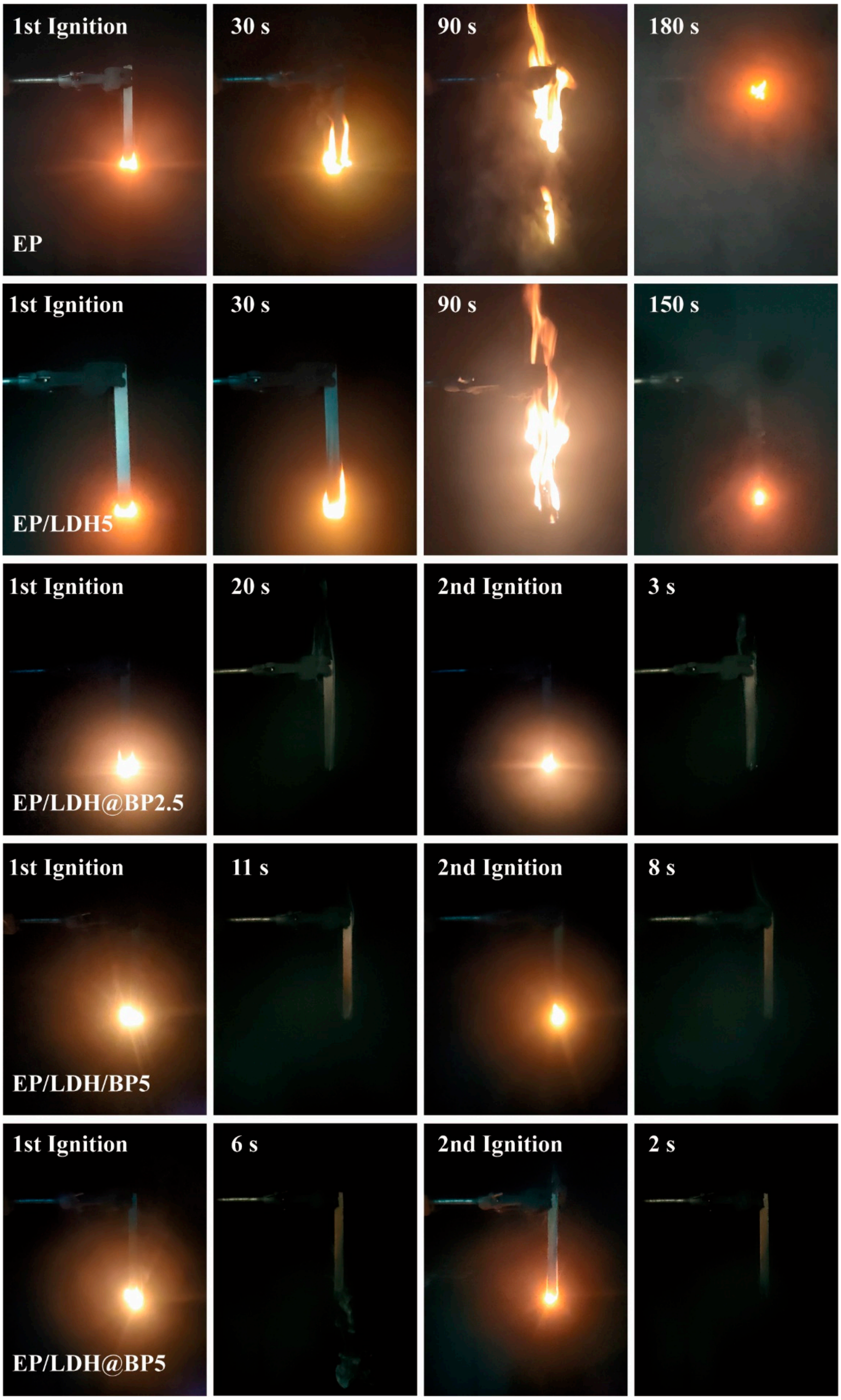

Figure S6. Digital photos of UL-94 vertical burning tests for EP, EP/LDH5, EP/LDH@BP2.5, EP/LDH/BP5, and EP/LDH@BP5 nanocomposites.

Table S1. The formulation of EP and other EP nanocomposites, and the corresponding results of LOI and UL-94.

Sample	DGEBA	LDH	LDH@BP	LDH/BP	DDM	UL-94	LOI	Dripping
EP	80	-	-	-	20	NR	24.2	Yes
EP/LDH5	76	5	-	-	19	NR	26.3	NO
EP/LDH@BP2.5	78	-	2.5	-	19.5	V-1	26.9	NO
EP/LDH/BP5	76	-	-	5	19	V-1	28.6	NO
EP/LDH@BP5	76	-	5	-	19	V-0	29.1	NO

References

1. Zhang H, Mao J, Li M, Cai Q, Li W, Huang C, et al. Design of h-BN@boronate polymer core-shell nanoplates to simultaneously enhance the flame retardancy and mechanical properties of epoxy resin through the interficial regulation. Composites Part A: Applied Science and Manufacturing. 2020;130.
2. Chu F, Ma C, Zhang T, Xu Z, Mu X, Cai W, et al. Renewable vanillin-based flame retardant toughening agent with ultra-low phosphorus loading for the fabrication of high-performance epoxy thermoset. Composites Part B: Engineering. 2020;190.
3. Liu C, Chen T, Yuan CH, Song CF, Chang Y, Chen GR, et al. Modification of epoxy resin through the self-assembly of a surfactant-like multi-element flame retardant. Journal of Materials Chemistry A. 2016;4(9):3462-70.
